# Manganese-enhanced MRI enables longitudinal tracking of transplanted stem cell viability in the murine myocardium

**DOI:** 10.1186/1532-429X-16-S1-O95

**Published:** 2014-01-16

**Authors:** Rajesh Dash, Aditya Subramanian, Yuka Matsuura, Il Suk Sohn, Ting-Yuan Yeh, Michael V McConnell, Joseph Wu, Phillip Yang

**Affiliations:** 1 Cardiovascular Medicine, Stanford University, Stanford, California, USA; 2 Engineering, Stanford University, Stanford, California, USA; 3 Cardiovascular Institute, Stanford University Medical Center, Stanford, California, USA

## Background

Stem cell therapy in the heart is limited by an inability to track transplanted cell survival. To address this limitation, we used human amnion-derived mesenchymal stem cells (hAMSCs), which exhibit longer in vivo survival, and Manganese (Mn2+)-Enhanced MRI (MEMRI), which enters live stem cells to augment T1 signal. We tested Mn2+ pre-labeling of hAMSCs in vitro and whether MEMRI would detect hAMSC survival in mouse myocardium in vivo.

## Methods

hAMSCs were isolated from human placentas after IRB consent. A subset of cells was transduced with a luciferase reporter gene. One group of hAMSCs was exposed to 1 μM doxorubicin (DOX) for 4 hrs, then incubated for 48 hrs. The hAMSCs (Healthy & DOX) were then labeled with increasing concentrations (0.1, 0.5, & 1 mM) of MnCl2 (Sigma, Inc) for 30 min. Bioluminescence (BLI) was performed after MnCl2 labeling. Cells were pelleted into Eppendorf tubes and in vitro 3T MRI was performed (SignaHDx, GE, Inc). For in vivo MEMRI, 0.25 × 106 Healthy and DOX hAMSCs were pre-labeled with 0.5 mM MnCl2 for 30 min, washed, and pelleted for direct injection into hindlimb & myocardium. Mice were immediately imaged using an FGRE-irP sequence: FOV4/ST 1 mm/TE min/TI 400 ms/NEX4. In vivo MEMRI was repeated 2 days later, after 250 μl of i.p. MnCl2.

## Results

**Figure 1:**
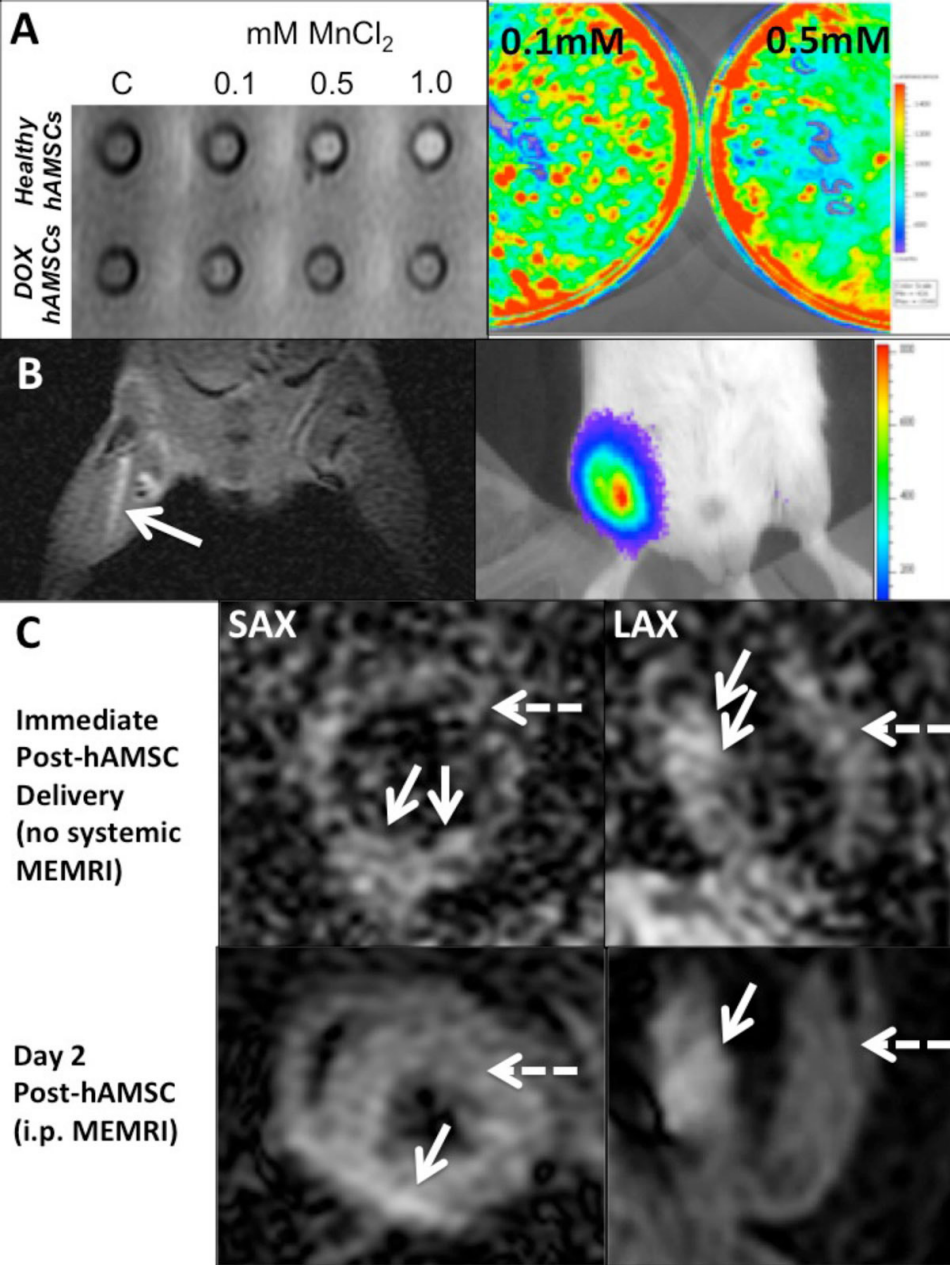
**A: (left): 0.5 mM MnCl2 was sufficient to increase the T1 signal of pre-labeled healthy hAMSCs, but injured/dead DOX hAMSCs failed to take up MnCl2; (right) BLI shows better survival of hAMSCs 48 hrs after incubation with either 0.1 mM or 0.5 mM MnCl2, compared to DOX hAMSCs**. 1B: (left) Hindlimb MRI showing positive MEMRI signal from pre-labeled hAMSCs in left hindlimb; (right) corresponding positive BLI signal from same hindlimb, confirming live hAMSCs. 1C: (top) short- (SAX) and long- (LAX) axis MEMRI of mouse heart immediately post-hAMSC delivery. Note the positive signal in the basal inferior wall (arrow) whereas simultaneous DOX hAMSC injection in anterior wall shows no MEMRI signal (dotted arrow); (bottom) 2 days after hAMSC delivery, i.p. MEMRI injection reveals intense uptake in basal inferior wall (healthy hAMSCs, arrow) with no uptake in anterior wall (DOX hAMSCs, dotted arrow).

0.5 mM MnCl2 increased the T1 signal and contrast-to-noise ratio (CNR) of healthy hAMSCs (ΔCNR 80 ± 2*) ~3x vs DOX hAMSCs (29 ± 2, *p < 0.05) due to BLI-verified cell dropout. BLI showed no reduction in hAMSC survival after Mn2+ labeling (Figure 1A). Hindlimb imaging showed increased MEMRI CNR (18 ± 3) and BLI signal from pre-labeled hAMSCs (1B). Cardiac MEMRI of Healthy hAMSCs showed positive signal immediately after delivery as well as 2 days later (1C). However, DOX hAMSCs showed no MEMRI signal in vivo.

## Conclusions

MEMRI successfully labels and tracks live, transplanted hAMSCs in the heart, enabling serial tracking of cell delivery and survival with no genetic pre-modification.

## Funding

NIH K08 (RD) NIH R01 (PY) Stanford CVI Seed Grant (RD).

